# Contralateral second dose improves antibody responses to a 2-dose mRNA vaccination regimen

**DOI:** 10.1172/JCI176411

**Published:** 2024-01-16

**Authors:** Sedigheh Fazli, Archana Thomas, Abram E. Estrada, Hiro A.P. Ross, David Xthona Lee, Steven Kazmierczak, Mark K. Slifka, David Montefiori, William B. Messer, Marcel E. Curlin

**Affiliations:** 1 Department of Occupational Health,; 2 Oregon National Primate Research Center, Division of Neuroscience, and; 3 Department of Molecular Microbiology and Immunology, Oregon Health & Science University, Portland, Oregon, USA.; 4 UCLA, Los Angeles, California, USA.; 5 Department of Pathology, Oregon Health & Science University, Portland, Oregon, USA.; 6 Department of Surgery, Duke University Medical Center, Durham, North Carolina, USA.; 7 Department of Medicine, Division of Infectious Diseases, Oregon Health & Science University, Portland, Oregon, USA.; 8 Program in Epidemiology, Oregon Health & Science University, Portland State University School of Public Health, Portland, Oregon, USA.; 9 Vaccine and Gene Therapy Institute, Oregon Health & Science University, Portland, Oregon, USA.

**Keywords:** Immunology, Infectious disease, Adaptive immunity

## Abstract

**BACKGROUND:**

Vaccination is typically administered without regard to site of prior vaccination, but this factor may substantially affect downstream immune responses.

**METHODS:**

We assessed serological responses to initial COVID-19 vaccination in baseline seronegative adults who received second-dose boosters in the ipsilateral or contralateral arm relative to initial vaccination. We measured serum SARS-CoV-2 spike–specific Ig, receptor-binding domain–specific (RBD-specific) IgG, SARS-CoV-2 nucleocapsid–specific IgG, and neutralizing antibody titers against SARS-CoV-2.D614G (early strain) and SARS-CoV-2.B.1.1.529 (Omicron) at approximately 0.6, 8, and 14 months after boosting.

**RESULTS:**

In 947 individuals, contralateral boosting was associated with higher spike-specific serum Ig, and this effect increased over time, from a 1.1-fold to a 1.4-fold increase by 14 months (*P* < 0.001). A similar pattern was seen for RBD-specific IgG. Among 54 pairs matched for age, sex, and relevant time intervals, arm groups had similar antibody levels at study visit 2 (W2), but contralateral boosting resulted in significantly higher binding and neutralizing antibody titers at W3 and W4, with progressive increase over time, ranging from 1.3-fold (total Ig, *P* = 0.007) to 4.0-fold (pseudovirus neutralization to B.1.1.529, *P* < 0.001).

**CONCLUSIONS:**

In previously unexposed adults receiving an initial vaccine series with the BNT162b2 mRNA COVID-19 vaccine, contralateral boosting substantially increases antibody magnitude and breadth at times beyond 3 weeks after vaccination. This effect should be considered during arm selection in the context of multidose vaccine regimens.

**FUNDING:**

M.J. Murdock Charitable Trust, OHSU Foundation, NIH.

## Abstract 2

In multi-dose vaccine regimens, use of the opposite arm for the second dose substantially increases antibody responses and the improvement increases over time.

## Introduction

In the 227 years since Edward Jenner demonstrated that inoculation with material from cowpox lesions could provide protection from symptomatic illness due to smallpox, vaccine research has advanced to the point of offering some of the most effective tools available to clinical medicine and public health for the prevention of infectious diseases. Nowhere is this more evident than in the remarkable concerted worldwide vaccine campaign in response to the COVID-19 epidemic, through which an estimated 5.5 billion people have received at least 1 dose of a COVID-19 vaccine (1). The US Food and Drug Administration has now approved vaccines for at least 33 infections in humans (2), and major efforts continue to develop successful vaccines against the most immunologically challenging pathogens for which effective vaccines have been elusive, such as HIV-1, tuberculosis, malaria, and hepatitis C (3–6). Meanwhile, the vaccine research field has diversified to include new vaccine platforms and modes of delivery (7–9) and an expanded range of targets such as various forms of cancer (10–13) and other noncommunicable diseases (14, 15).

Currently available vaccines against infectious diseases confer protection largely by eliciting neutralizing antibodies (NAbs) that sterilize or reduce the pathogenic effect of infectious agents, although several vaccines are also designed to elicit cytotoxic T cell responses, which are recognized as playing a role in reducing pathogenesis in many settings. While certain vaccines, such as the yellow fever vaccine, are given as a single dose, most vaccine regimens rely on a prime-boost strategy in which multiple doses are given over time to elicit optimal immune responses. Until now, little has been known about the impact of vaccination site when administering vaccine boosters. For standard vaccines, arm selection has not been regarded as immunologically consequential (16, 17), and standard recommendations on vaccine placement are based on considerations such as sufficient muscle mass relative to vaccine volume, particularly in pediatrics (18), avoidance of nearby structures, such as the sciatic nerve and the subacromial bursa (19, 20), and patient comfort (21). However, according to the current paradigm for development of adaptive immune responses after localized exposure, DCs take up antigen from the site of exposure and transit along afferent lymphatic pathways to regional lymph nodes, then to the lymph node paracortex, where presentation to antigen-specific B cells and follicular T helper cells initiates the development of adaptive humoral and cellular responses. Studies using dye injection, cellular tagging with immunofluorescent markers, intravital microscopy, and other methods have demonstrated a regulated system of regionally convergent lymphatic flow and cellular traffic from peripheral sites to corresponding local draining lymph nodes (22–27), which appear to serve a dual role as both sites for antigen presentation and as sentinels along the path to the thoracic duct, presenting a barrier to systemic spread of invading pathogens (28, 29).

Localization of antigen presentation to specific lymph node environments based on the original site of exposure raises the possibility that some aspects of adaptive immunity after multiple sequential vaccinations could be sensitive to vaccination site. We are aware of only 4 studies addressing this question (30–33). A study in mice reported that in prime-boost vaccination with influenza HA, ipsilateral and contralateral boosting resulted in different frequencies of antigen-specific clonal IgGs from secondary germinal center B cells, though total antigen-specific IgG levels were similar in each group (33). Iro reported that ipsilateral vaccination resulted in lower *Haemophilus influenzae* type b–specific IgG among 509 infants receiving DTap-IPV-Hib (diphtheria and tetanus toxoids and acellular pertussis adsorbed, inactivated poliovirus and haemophilus B conjugate vaccine) vaccination in the context of neonatal vaccination series at 2, 3, and 4 months of age (31). However, most recently, Ziegler reported higher NAb titers and levels of spike-specific CD8^+^ T cells following ipsilateral second dosing in individuals randomized to receive a second mRNA-based COVID vaccine in either the ipsilateral or contralateral arm (32). Given the limited and inconsistent information on the effect of booster dose placement on immune responses in humans, we examined antibody responses in adults returning for the second dose of an initial 2-dose COVID-19 vaccine regimen based on whether they received the second vaccination in the ipsilateral arm or the contralateral arm relative to the initial vaccination.

## Results

### Enrollment and demographics.

**Figure 1:**
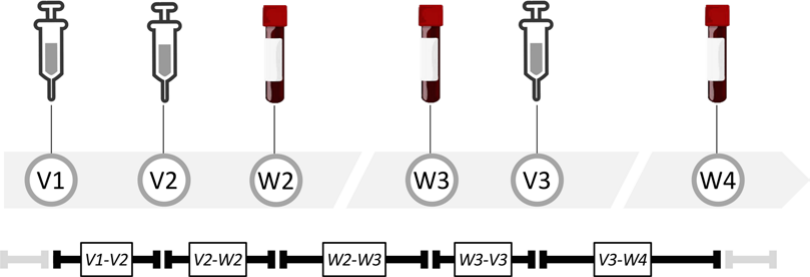
Study visits and procedures. Time line of vaccinations, arm randomization, and study visits for blood collection. Vaccination and visit time points are represented by V and W, respectively.

Among 2,016 participants in the OHSU COVID-19 Serology Study (C19 cohort) receiving a second vaccination, 36 did not have vaccination site recorded, and 391 were lost to follow-up. Among the remaining 1,589 (unselected analysis group), 1,075 were approached for enrollment in the arm-selection substudy, 958 consented, and 947 were seronegative at baseline (enrolled analysis group). In this group, the mean age was 44.5 years (range 24–84, IQR 19.0); 73% were female and 23% were male. Within this group, the same-arm (*n* = 507) and opposite-arm (*n* = 440) subgroups were closely matched with respect to age, sex, and relevant time intervals between vaccination and sampling (Figure 1, Table 1 and Supplemental Figure 2; supplemental material available online with this article; https://doi.org/10.1172/JCI176411DS1). In the enrolled analysis group, 940 individuals returned for study visit 2 (W2), 636 returned for W3 and were nucleocapsid-seronegative at this visit, and 317 returned for W4, had a recorded vaccine dose 3 (V3), and remained nucleocapsid seronegative at W4 (Supplemental Figure 1).

### Antibody titers: enrolled analysis group.

**Figure 2:**
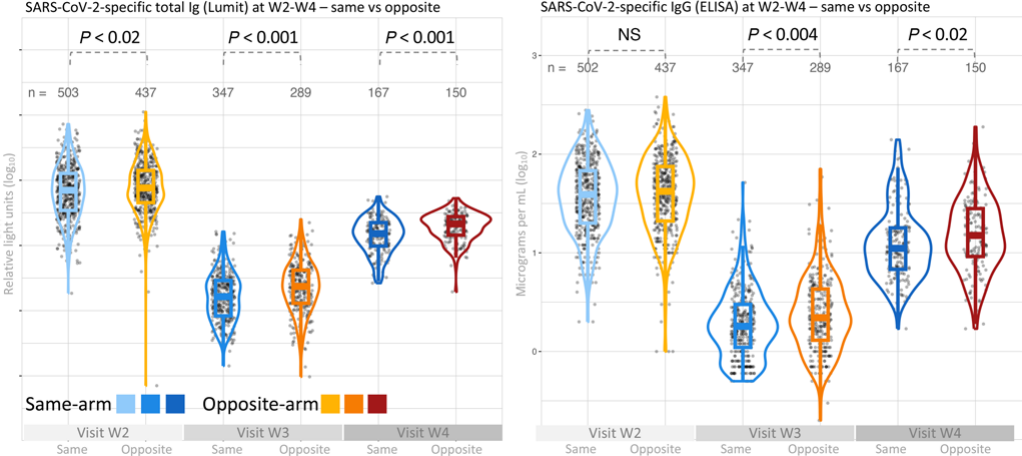
Serological responses to vaccination after contralateral- and ipsilateral-arm boosting (enrolled analysis group). Comparison of serum SARS-CoV-2–specific total Ig (left panel) and IgG (right panel) in 947 individuals receiving the second vaccine dose in the same or opposite arm relative to the first vaccine dose. Measurements were performed at visit W2 (shortly after V2), visit W3 (before V3), and at visit W4 (several months after V3). Ig levels were determined by the Lumit assay and IgG measured by ELISA. All antibody levels are shown as log_10_ transformed values. Significance was determined by 2-tailed *t* test

We first measured total receptor-binding domain–specific (RBD-specific) antibodies using a bioluminescence-based assay detecting IgM, IgA, and IgG (Lumit Dx SARS-CoV-2 Immunoassay). Overall mean RBD-specific responses reached 712 RLU approximately 2.5 weeks after V2, waned to a mean of 20 RLU at 8 months, and rose to a mean of 164 RLU by 1.1 years, following V3 boosting (Figure 2). Among those receiving contralateral boosting at V2, there was a progressive relative increase in antibody-response titers across these time points in comparison with those receiving ipsilateral boosting, with titers 1.2-fold higher at W2 (*P* = 0.02), 1.4-fold higher at W3 (*P* < 0.001), and 1.4-fold higher at W4 (*P* < 0.001) (Table 2). We next measured SARS-CoV-2 RBD–specific IgG in these same groups using an ELISA assay standardized to report in absolute antibody concentrations (μg/mL). Overall IgG antibody titers followed a similar response profile across the 3 time points, from 38 μg/mL to 2 μg/ml to 16 μg/ml at these time points. Here, W2 titers were similar between arm groups, but higher titers were seen in the contralateral subgroup at W3 (1.2-fold higher) and W4 (1.3-fold higher) across the sampling period of approximately 1.1 years (Table 2 and Figure 2).

### Antibody titers: matched-pair analysis group.

**Figure 3:**
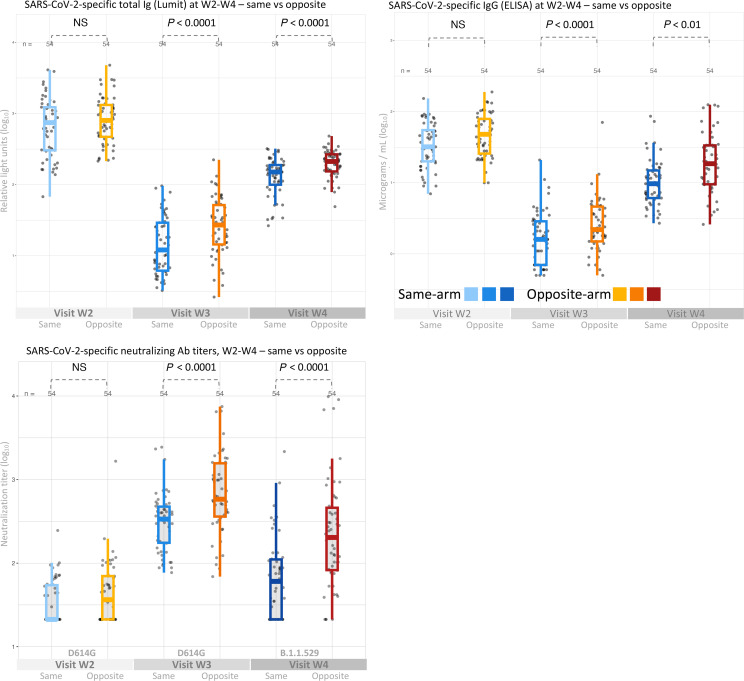
Antibody responses in matched pairs. Comparison of serum SARS-CoV-2–specific total Ig (upper left), IgG (upper right), and neutralization titer against early COVID-19 strain D614G and Omicron strain B.1.1.529 (lower left) in 54 matched pairs (*n* = 108) receiving the second vaccine dose in the same or opposite arm relative to the first vaccine dose. Measurements were performed at W2 (shortly after V2), W3 (before V3), and W4 (several months after V3). Ig levels were determined by the Lumit assay, IgG measured by ELISA, and neutralization titer by pseudovirus neutralization assay. All antibody levels are shown as log_10_-transformed values. Significance was determined by 2-tailed paired *t* test.

We next compared antibody titers in a subset of 108 participants (54 pairs) matched by age, sex, and relevant vaccination and sampling time intervals. In this subset, SARS-CoV-2–specific Ig and IgG followed a similar pattern of higher responses in those receiving contralateral vaccination, with 1.7- and 1.5-fold increases at W3 (*P* ≤ 0.02) and 1.3- and 1.7-fold increases at W4 (*P* < 0.007), respectively (Table 2). We next tested neutralization against a pseudotyped virus harboring the spike S1 domain D614G mutation closely approximating the initial SARS-CoV-2 virus and B.1.1.529, an Omicron variant first reported in South Africa in November 2021, roughly 9 months after immunization at V2 occurred in this cohort. In this group, neutralization titers against a pseudovirus representing the D614G early epidemic strain were not significantly different in the 2-arm subgroups at W3, but were roughly 2-fold higher in the contralateral group at W4 (*P* < 0.001). Most notably, neutralization titers against a pseudovirus representing the “future” Omicron strain B.1.1.529 arising later in the epidemic were 3.5- to 4-fold greater in the contralateral group (*P* < 0.001) (Table 2 and Figure 3).

### Exploratory analyses.

In additional analyses, we examined the effect of arm selection at V2 in an unselected “real-world” population of 1,568 adults irrespective of infection status and receipt of additional boosters beyond V2 (unselected analysis group). In this group, qualitatively similar results showing a contralateral vaccination advantage were obtained (Supplemental Figure 3). We next examined vaccine-induced immunity in a subset of 139 individuals with serological evidence of COVID-19 infection prior to W3. A trend toward contralateral arm advantage was also seen in these individuals, suggesting that arm alternation could improve long-term responses even in those with subsequent natural infection (Supplemental Figure 4). To understand the effect of arm selection at doses beyond V2, we examined responses by arm-usage pattern in those receiving 3 vaccine doses. In this subanalysis, trends indicate that the key differentiator is arm alternation at initial immunological exposure (i.e., between V1 and V2), while arm choice at subsequent doses has little additional effect (Supplemental Figure 5). To shed additional light on the time dependence of the contralateral arm effect, we examined antibody titers at W2 by arm group as a function of time. We found that responses were consistent with a crossover point at approximately 2 weeks after V2, beyond which contralateral vaccination increases responses relative to ipsilateral vaccination at V2 (Supplemental Figure 6). Finally, to better understand the mechanism for improved neutralization resulting from arm alternation, we examined the ratio of neutralization to binding antibodies. This “quality ratio” was higher in the opposite-arm group, suggesting that gains in neutralization achieved by this strategy arise from increases in both magnitude and affinity of the antibody response (Supplemental Figure 7).

## Discussion

In this study, we found that in adults presenting for an initial vaccine series with an RNA-based COVID-19 vaccine, those receiving the second vaccination in the contralateral arm relative to the first dose developed significantly higher SARS-CoV-2–specific serum antibodies. This effect was reflected in total RBD-specific Ig, RBD-specific IgG, and neutralization titers, was independent of sex and age, and was observed in assays against both early strains closely related to the vaccine strain (Wuhan-Hi-1 and D614G) and against Omicron BA1.1.529, a variant appearing later in the epidemic, to which the cohort had no exposure at the time of vaccination. In our setting, age, sex, prior exposure, and interval between first and second vaccinations influenced antibody levels, but these effects became insignificant over time with repeated exposure (our unpublished observations). In contrast, the relative effect of arm alternation increased over the period of approximately 1.1 years between second vaccination and final sampling (Table 1). The placement effect we observed appears to be associated with initial vaccination and first boost, while the role of placement at the time of subsequent vaccinations is uncertain (Supplemental Figure 5).

It is commonly expected that immune responses are indifferent to ipsilateral versus contralateral boosting as part of a multidose intramuscular vaccine regimen. However, in a study by Iro et al., among 509 children receiving diphtheria–tetanus–acellular pertussis–inactivated polio–*Haemophilus influenzae* type b combined vaccine as part of routine infant vaccination, those randomized to receive the second dose in the contralateral leg developed significantly greater geometric mean antibody concentrations against selected vaccine components at 5, 12, 13, and 24 months (31). In contrast, Ziegler recently reported that in 303 adults randomized to receive a second dose of BNT162b2 on the ipsilateral or contralateral side, spike-specific IgG levels did not differ between groups, but neutralizing activity and spike-specific CD8 were significantly lower in the contralateral group at 2 weeks after the second dose (32), and suggested that ipsilateral dosing may be preferred.

We hypothesize that the discrepancy between our results and those of Ziegler are related to the timing of their assessment, which took place at 2 weeks. This would be very early in the process of immune response maturation, at a point when preformed germinal centers on the side of initial vaccination would likely provide an initial head start in those receiving ipsilateral boosting. However, memory B cell expansion and affinity maturation proceed for many months after vaccination (34, 35). In our study, the first post-boost sampling occurred at a median of 20.4 days (IQR 7 days, Supplemental Figure 2). A slight improvement with contralateral boosting at this time resolved into a clear long-term advantage by 8 months and continued to increase beyond 1 year after vaccination. We therefore speculate that there is a crossover time at some point after 2 to 3 weeks, at which time contralateral boosting becomes superior to ipsilateral boosting (Supplemental Figure 6).

The mechanisms by which site selection may influence humoral immunity are poorly understood, and we are only aware of one prior animal study addressing this question. Kuraoka et al. showed that when mice are immunized with influenza HA and later boosted on either the same or the opposite side, there was a significant increase in HA-specific serum IgG antibodies in both groups, with a nonsignificant trend toward increased levels in the ipsilateral group (33). Enumeration of HA-specific B cells within draining lymph node secondary germinal centers by flow cytometry, cell sorting, and single-cell culture assays revealed substantial increases in both groups, although the frequency of HA-specific clonal B cells and measures of antibody affinity were significantly greater in the ipsilateral group. Interestingly, ipsilateral boosting was associated with more efficient recruitment of the progeny of vaccination-induced primary germinal center B cells to secondary germinal centers, while contralateral boosting was associated with a greater representation of newly activated naive B cells. This would imply that site alternation might ultimately result in a larger pool of memory B cells capable of mounting a recall response.

The results presented here are noteworthy because antigen-specific antibody titers are a recognized correlate of protection from many pathogens (36) and interventions capable of increasing antibody responses by a factor of 4-fold, as we saw in this study for neutralizing responses at W4, would be potentially impactful in a variety of settings. In the COVID study, a major phase 3 COVID-19 vaccine efficacy (VE) trial (37), protection from COVID-19 was highly correlated with day-57 NAb titers, with VE estimated to be 51%, 78%, 91%, and 96%, for ID_50_ values of undetectable, 10, 100, and 1,000 IU_50_/mL, respectively. In this context, increasing NAb titers from undetectable to even the lowest measured ID_50_ corresponded to a 2.2-fold increase in risk reduction, while an increase in log_10_ NAb titer from 1.66 to 2.27 corresponded to a 1.8-fold decrease in likelihood of acquiring COVID-19 (38). In the search for an effective HIV vaccine, serological responses capable of mediated sterilizing immunity prior to establishment of viral latency within infected cells is particularly desirable, and it is likely that antibody titers will be a critical end point in any eventually successful vaccine. In RV144, the only study to date to demonstrate any efficacy for the prevention of HIV, peak VE at 12 months was estimated to be 60%, but overall VE at 42 months was unfortunately only 31.2%, which was below the prespecified threshold to pursue further development (39, 40). IgG binding to variable regions 1 and 2 of the HIV envelope protein was identified as a likely correlate of protection (41). However, no data are available on the effect of arm selection in this study.

This study has important limitations. Our results could have been affected by bias introduced related to participant choice during arm selection or other unrecognized factors such as vaccine batch effects. However, our study involved a relatively large cohort enrolled over a relatively long period, and arm groups were balanced, reducing the likelihood that chance factors or systematic bias could be entirely responsible for the effects observed here. We measured neutralization titers by the same methods as other major studies in this area, allowing direct comparison with other important COVID-19 clinical data sets. We examined responses in adults receiving a homologous prime-boost mRNA-based vaccine by the intramuscular route, and our results do not address alternative routes of immunization such as oral vaccines, unconventional dosing practices such as contemporaneous multisite vaccination, and qualitatively dissimilar vaccine platforms, such as protein-based, vector-based, and live-attenuated vaccines. Responses to live-attenuated vaccines, in particular, might not be sensitive to vaccine placement if priming occurs at multiple lymphoid centers through dissemination of a replicating vector. Because pathogen-specific antibody titers are an accepted measurable correlate of protection from infection, we focused on serological responses. However, we do not address cellular immunity, which is thought to protect from serious illness after established infection in a variety of settings.

Prospective randomized studies will be necessary to clarify the impact of administration route, timing vaccine platform, prior exposure, and vaccinations after the initial series. Future studies should include assessments of cellular immunity, enumeration of antigen-specific memory B cells, and sampling at time points between 2 weeks and several months after vaccination to provide further insights into the underlying mechanisms of the vaccine site effect described here. Studies in children are also needed, since several prime-boost vaccine regimens are essential components of pediatric care, and immune responses may differ in children.

In conclusion, contralateral placement of boosters appears to substantially increase vaccine-specific antibody responses following mRNA COVID-19 vaccination, at least in some settings. Further investigation is needed to understand the relevance to other vaccines, and the effect of placement should also be studied in children, given that vaccines are an essential component of routine pediatric care. If confirmed in future studies, placement effect could have important implications for vaccine administration during clinical care and for the conduct and interpretation of vaccine-related research.

## Methods

### Study population.

The OHSU COVID–19 Serology Study (C19 cohort) is a longitudinal observation cohort of adult male and female healthcare workers presenting for initial COVID-19 vaccination at OHSU Occupational Health between December 16, 2020, and March 2, 2021. Eligibility criteria for participation in the C19 cohort included age greater than 18, intention to receive the first dose of the Pfizer-BioNTech vaccine (BNT162b2), absence of contraindication to phlebotomy, and ability to provide informed consent.

### Vaccination and study visits.

During the observation period, workforce members presented for up to 3 standard vaccine doses (V1, V2, and V3) provided in the Occupational Health clinic. At each vaccination visit, individuals received a standard 0.3 mL dose of the Pfizer-BionNtech COVID-19 vaccine BNT162b2 in the left or right deltoid muscle, according to manufacturer instructions. Study participants were also seen at 3 study visits for sampling: visit W3 soon after V2, visit W3 occurring before third-dose boosting, and at visit W4, occurring after V3 (Figure 1 and Table 1). At each sampling visit, approximately 8 mL of peripheral venous blood was collected by standard phlebotomy in red-topped vacuum tubes, centrifuged at 1,600*g* for 10 minutes, and frozen at –20°C until use.

### Arm selection, matching, and blinding.

All participants with evaluable data were included in this study. Prior to the second vaccine dose, a subset of 1,075 participants was invited to consider vaccination in either the ipsilateral or contralateral arm relative to the initial vaccine, according to a randomized list. Actual vaccination site was left to participant preference at the time of vaccination, which occurred separately without involvement by the study team. At the time of laboratory assessment, a matched subset of 54 participants receiving ipsilateral boosters and 54 receiving contralateral boosting at V2 was selected for NAb assays (see below). Matching was performed by algorithm so as to minimize a weighted score accounting for differences in sex, age, and time intervals between vaccine doses V1 and V2, V2 and sampling at W3, and V3 and sampling at W4. Samples were coded with nonsequential randomly generated 5-digit identification codes at study initiation. This number was used for sample labeling, and all laboratory work was performed on deidentified samples by staff blinded to participant identity and group allocation.

### SARS-CoV-2–specific serum total Ig assays.

SARS-CoV-2 spike protein–specific total antibody titers were measured using the Lumit Dx SARS-CoV-2 Immunoassay (Promega Corp.) (42). The test detects total Ig specific for the RBD antigen within the SARS-CoV-2 spike protein. The assay utilizes bioluminescence complementation technology to generate a luminescent signal that is detected on a luminescent-capable microtiter plate reader when SARS-CoV-2 antibodies are present. Samples were processed according to manufacturer instructions. CoV-2-SmBiT and CoV-2-LgBiT were added to 96-well plates. Positive controls, negative controls, or 10 μL serum was added to wells. Plates were incubated at ambient temperature for 30 minutes, after which Lumit Dx Detection Reagent was added and luminescence was measured using a luminometer. Preliminary assay results were calculated as the ratio of test sample to mean calibrator luminescent signal. Samples exceeding the upper limit of detection were diluted as necessary by factors of 15, assays were repeated, and true concentrations backcalculated. Results were recorded as RLUs.

### SARS-CoV-2–specific IgG assays.

SARS-CoV-2 RBD–specific and nucleocapsid-specific IgG-binding antibody levels were determined by ELISA as previously reported (43); 96-well ELISA plates (catalog 9018, Corning) were coated with 100 μL of either recombinant SARS-CoV-2 Wuhan-Hi-1 RBD (catalog 230-30162, Ray Biotech) or nucleocapsid protein (catalog 40588-V08B, Sino Biologicals) at a concentration of 1 μg/mL in PBS, incubated overnight at 4°C, and stored at –20°C until use. Plates were thawed at room temperature (RT), inverted over a sink and tapped on paper towels to remove excess antigen in solution, and blocked for 1 hour at RT with 5% nonfat dry milk (Kroger) prepared in PBS-Tween containing 0.05% Tween (dilution buffer) and washed once with PBST containing 0.05% Tween (wash buffer). Heat-inactivated serum samples were serially 3-fold diluted in plate wells using 100 μl dilution buffer. Plates were incubated at RT for 1 hour, and 50 μL of 10% hydrogen peroxide was added to each well and incubated for 30 minutes at RT. The plates were washed 3 times with wash buffer, and 100 μL of 1:2,000 dilution of antihuman IgG-HRP (catalog 555788, BD Biosciences — Pharmingen) detection antibody was added. Plates were incubated at RT for 1 hour and washed 3 times with wash buffer; 100 μL of colorimetric detection reagent containing 0.4 mg/mL of o-phenylenediamine and 0.01% hydrogen peroxide in 0.05M citrate buffer (pH 5) was added, and the reaction was stopped after 20 minutes by the addition of 100 μL of 1-M HCl. OD at 490 nm was measured using a VersaMax ELISA plate reader (Molecular Devices). A standard curve generated using SARS-CoV-2–reactive monoclonal antibody ABMX-002 (catalog 10–2005, Abeomics) was included in duplicate on every RBD plate, and an internal positive control serum standard was included in every nucleocapsid ELISA plate to normalize ELISA titers between experiments. Antibody titers were determined by logarithmic transformation of the linear portion of the curve, with 0.1 OD units used as the end point before converting to final values. Results were reported as μg/mL.

### Strain-specific pseudovirus neutralization assays.

SARS-CoV-2–specific serum NAbs were measured using a validated pseudovirus-based assay measuring reductions in luciferase reporter gene expression after a single round of infection with either SARS-CoV-2.D614G or SARS-CoV-2.BA1.1.529 spike-pseudotyped virus in 293T/ACE2 cells, as previously reported (38). Briefly, serum samples were heat inactivated for 30 minutes at 56°C prior to use. Spike-pseudotyped virus was prepared by transfection in 293T cells (human embryonic kidney cells in origin; ATCC, catalog CRL-11268) using a lentivirus backbone vector, a spike-expression plasmid, a TMPRSS2 expression plasmid, and a firefly Luc reporter plasmid. A pretitrated dose of pseudovirus was incubated with 8 serial 5-fold dilutions of serum samples (1:30 start dilution) in duplicate in 96-well, low-evaporation, sterile, flat-bottom culture plates (Corning) for 1 hour at 37°C prior to adding 293T/ACE2 cells. One set of 8 wells received cells and virus (virus control), and another set of 8 wells received cells only (background control), corresponding to technical replicates. Luminescence was measured after 66 to 72 hours of incubation using Promega 1X lysis buffer (Promega, catalog E1531) and Bright-Glo luciferase reagent (Promega, catalog E2650). Neutralization titers were calculated as the inhibitory dilution of serum samples at which relative luminescence (measured as RLU) was reduced by either 50% (ID_50_) or 80% (ID_80_) compared with virus controls after subtraction of background RLUs.

### Statistics.

Visit data and laboratory assay data were recorded in spreadsheets at the time of collection and entered into a custom-developed, access-controlled, audit-tracked relational database. All data for analysis were retrieved from the database by structured queries in SQL. Analysis included all C19 serology participants receiving vaccinations at V1 and V2 who had complete information on vaccination site, had no evidence of prior COVID infection at W3, and provided samples on at least 1 return visit at either W3 or W4. Analyses were performed on all available subjects and on a case-control subset selected as described for NAb studies. Binding and NAb titers below the lower limits of detection were left-censored at LoD/2^1/2^, and analyses were performed on log-transformed values. Sex proportions in each group were compared with the 2-proportions *z* test, and continuous variables were compared by group using the *t* test (all subjects) or paired *t* test (matched pair subset). All statistical tests were 2 sided and reported using a *P* value significance threshold of 0.05. Analyses were performed using R version 4.2.1 in RStudio 2022.07.0.

### Study approval.

This study was approved by the Oregon Health & Science University Institutional Review Board as a nontrial observational study. All participants provided written, informed consent.

### Data availability.

Deidentified data used in this study are available by request. The code used in data analysis may be accessed at https://github.com/mecurlin/Vaccine-arm-selection.git (1 parent a73ec48 commit ID 4499b51). Values for all data points in graphs are reported in the Supporting Data Values file.

## Author contributions

SF performed data management, data analysis, and manuscript review. AT performed ELISA assays and manuscript review and editing. AEE performed sample management, sample processing, and manuscript review. HAPR provided recruitment and enrollment, sample collection, data analysis, and manuscript review. DXL performed data analysis and manuscript review. SK performed Promega-binding antibody assays and manuscript review and editing. MKS performed ELISA assays, interpretation of data, and manuscript review and editing. DM performed NAb assays and manuscript review and editing. WBM performed sample collection and processing, data interpretation, and manuscript review and editing. MEC conceptualized the study and performed recruitment, enrollment, sample collection and processing, and data analysis and interpretation and drafted the manuscript.

## Supplementary Material

Supplemental data

ICMJE disclosure forms

Supporting data values

## Figures and Tables

**Table 2:**
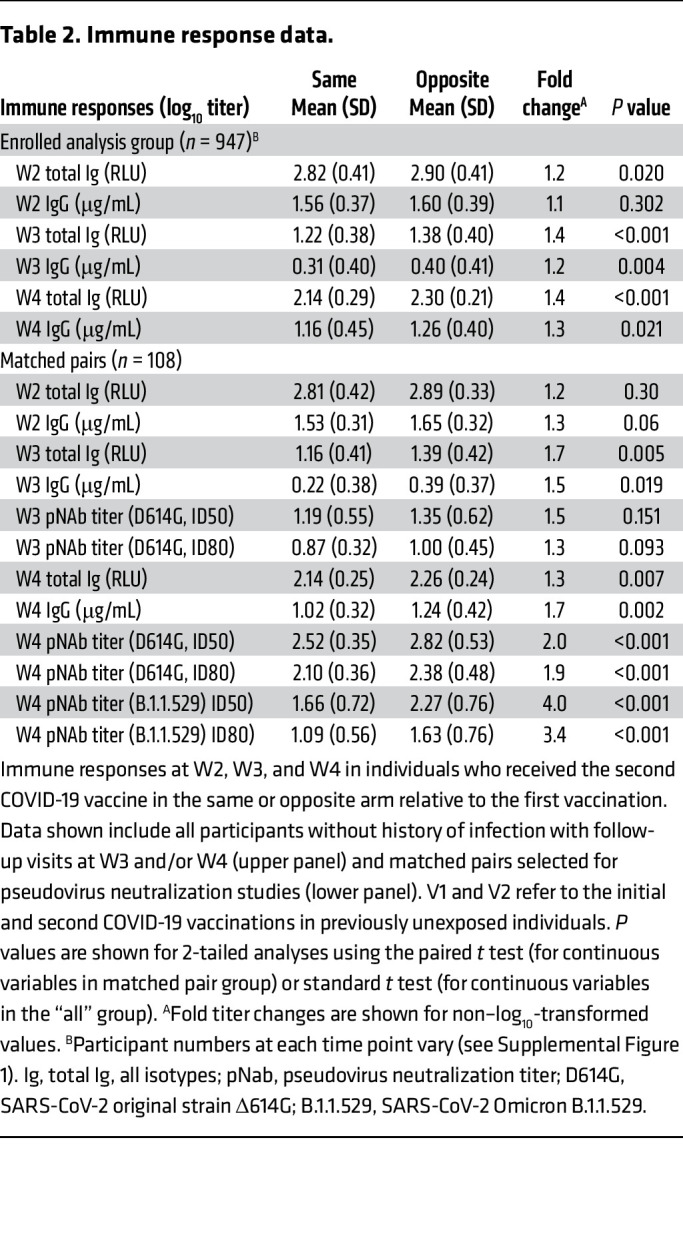
Immune response data.

**Table 1:**
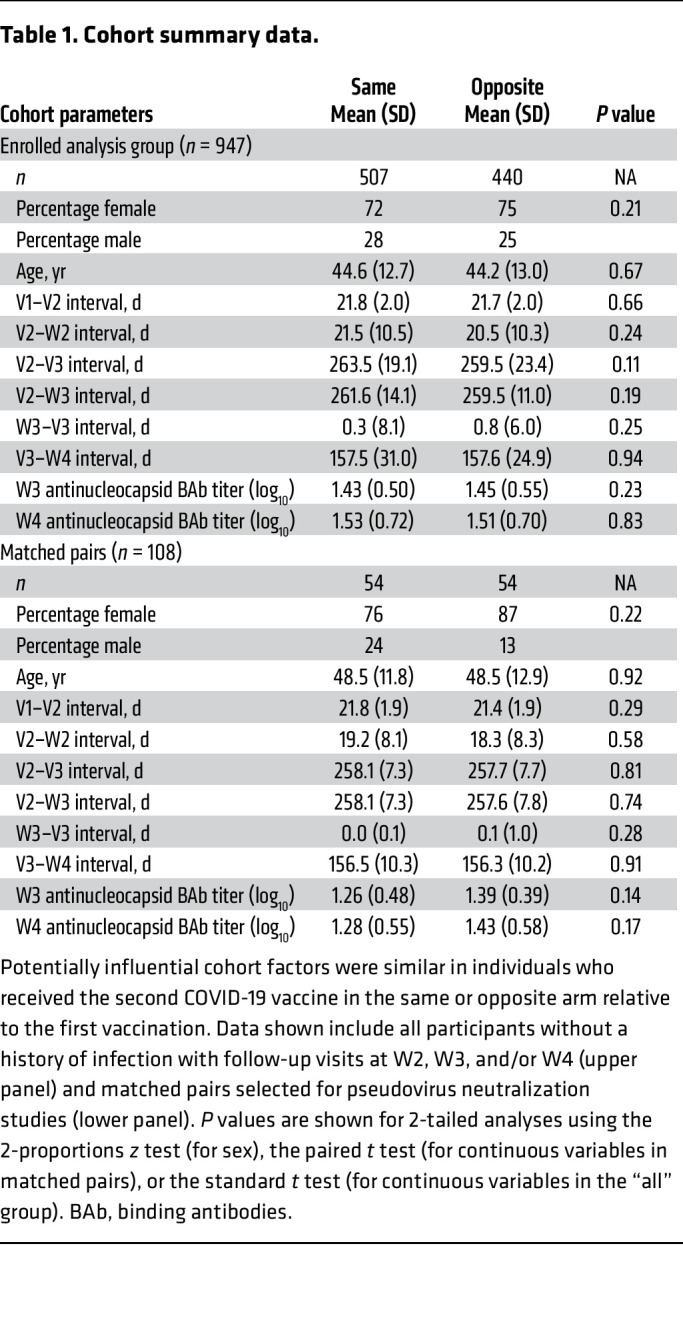
Cohort summary data.

## Version 1 01/16/2024

In-Press Preview

## Version 2 03/15/2024

Electronic publication
