# Supplementary material for: Contralateral second dose improves antibody responses to a 2-dose mRNA vaccination regimen
Source: J Clin Invest. 2024 Jan 16;134(6):e176411. doi: 10.1172/JCI176411 (PMC10940087; doi:10.1172/JCI176411)
Supplement: Supplemental data [file jci-134-176411-s006.pdf]

**Enrollment and cohort composition**: Total observational cohort size, group composition and breakdown by available data, enrollment, exclusions, vaccination status, natural infection status.

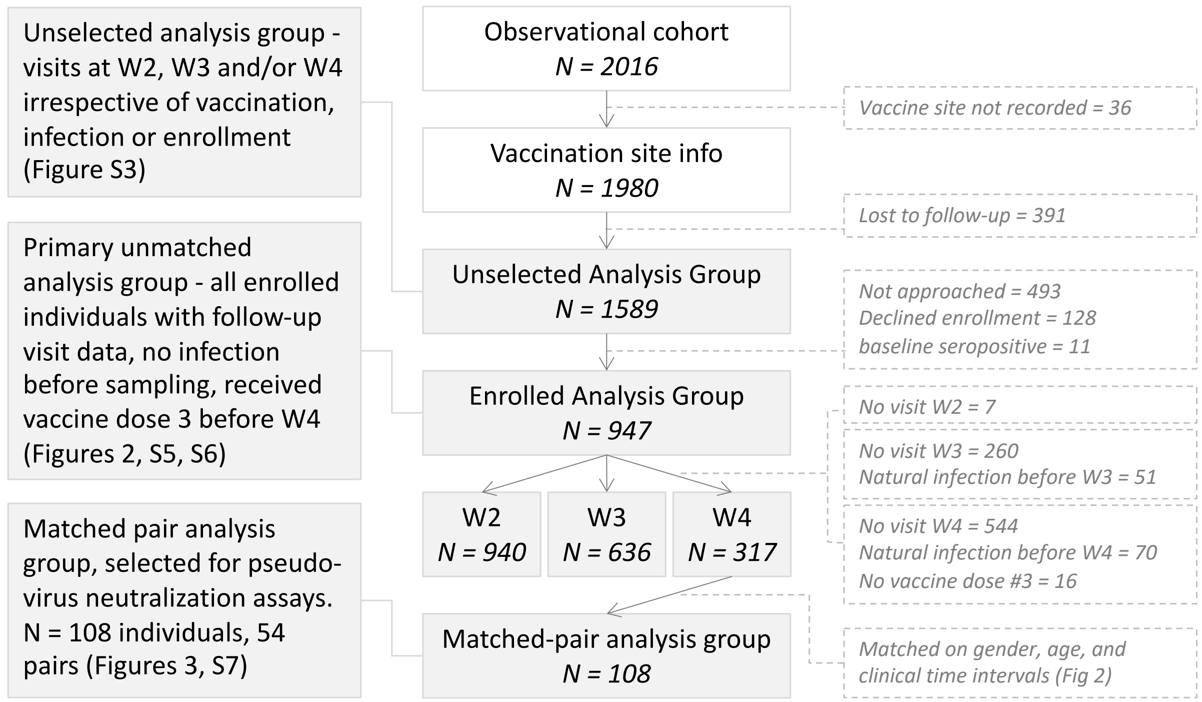


**Figure S1 – Cohort enrollment and group sizes.** Group sizes (center column), selection/exclusion criteria (right column) and analyses (left column). Vaccination and visit timepoints are represented by “V_”, “W_” respectively (see Figure 1)

**Baseline characteristics by group (Enrolled Analysis Group)**: To examine factors potentially influencing immune responses differently between groups, we compared time and vaccination intervals and age distribution between same-arm and opposite arm groups. These measures were closely matched in these two groups (**Table 1 and Figure S2**) suggesting that the groups are comparable.

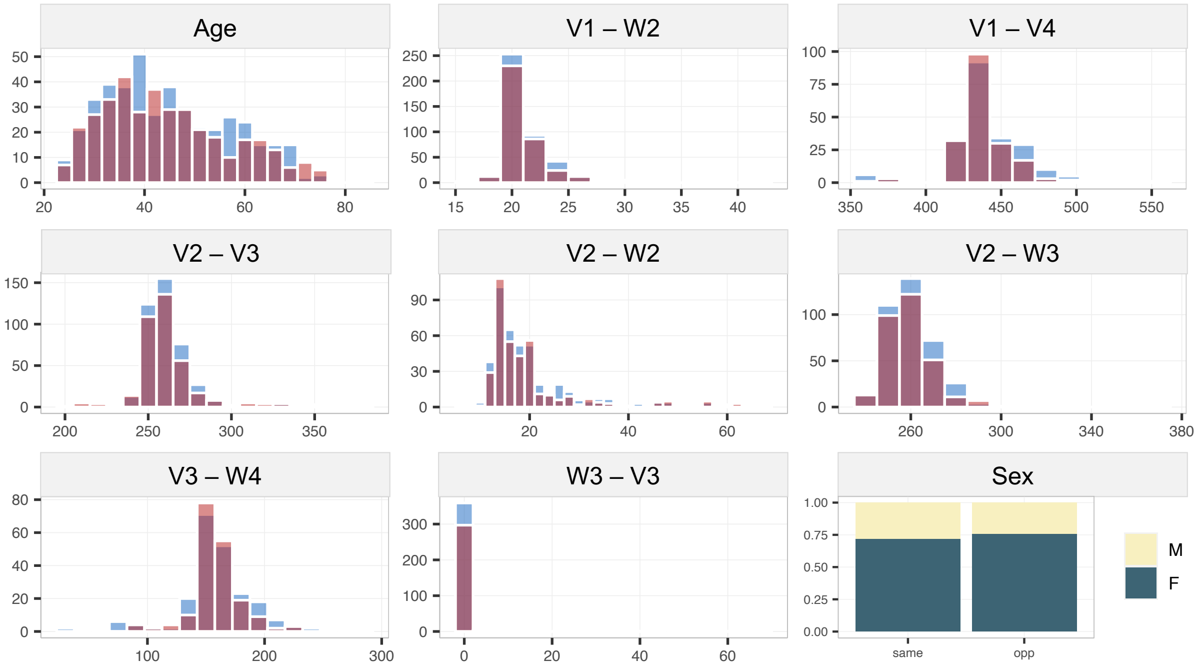


**Figure S2. Comparison of time intervals, age and sex by arm group (Enrolled Analysis Group)**. Time intervals area shown in days, age is shown in years. Vaccination and visit timepoints are represented by “V_”, “W_” respectively (see Figure 1). **Interpretation**: same and opposite arm groups are similar with respect to clinical time intervals and age distribution. Same Opposite

**Effect of arm choice in an unselected population (Unselected Analysis Group):** To understand the effect of arm selection at V1 and V2 in a "real-world" unselected population we repeated analysis of Ig and IgG responses at W3 and W4 in all cohort patients with available data regardless of infection status or receipt (or lack of receipt) of boosting at V3 (N = 1225). Qualitatively similar results were obtained, indicating a significant and persistent long-term increase in SARS-CoV-2-specific antibody titers in those receiving boosting in the opposite arm relative to initial vaccination. As expected, the magnitude of the effect in this group is smaller, likely reflecting the impact of confounding variables (**Figure S3**).

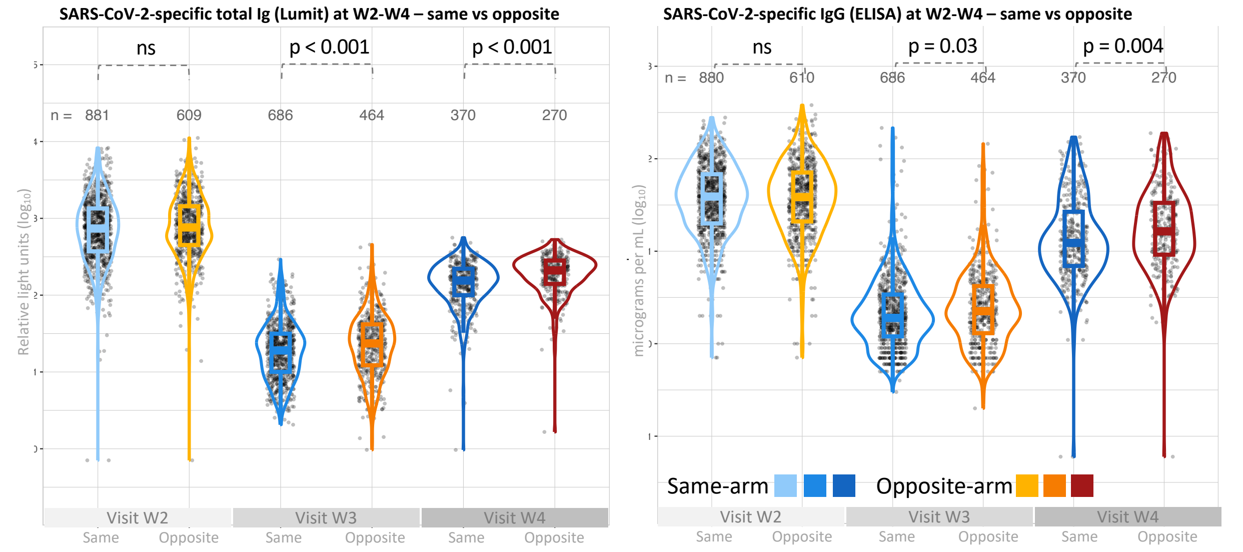


**Figure S3. SARS-CoV-2-specific total Ig and IgG at W3 and W4, by arm group in unselected participants (Unselected Analysis Group).** Analysis of the effect of arm selection at V1 and V2 irrespective of infection history and receipt of V3 boosting. Left panel: Total SARS-CoV2 specific Ig titer ($log_{10}$ RLU) by Lumit assay. Right panel: SARS-CoV-2-specific IG by ELISA ($log_{10}$ ug/ml) by ELISA at W3 and W4 in persons receiving V2 in the same arm or opposite arm relative to V1. Vaccination and visit timepoints are represented by "V_", "W_" respectively (see Figure 1). Significance determined by two-tailed T-test. **Interpretation**: Contralateral boosting confers a measurable immunologic advantage in an unselected real-world population regardless of natural infection and 3rd-dose boosting history.

**Arm selection in participants with natural infection:** To examine the effect of arm selection on immune responses after boosting in individuals with history of natural COVID-19 infection, we performed analyses including only individuals positive for SARS-CoV-2 nucleocapsid-specific antibodies at W3. Altogether, 139 participants had positive NC titers at W3, including 75 in the same-arm group and 63 in the opposite arm group. Qualitatively similar results were seen in this subgroup, with higher SARS-CoV2-specific Ig and IgG responses in those receiving vaccination in the opposite arm at V2. This trend was seen in all groups but only reached significance at W4 in measures of total Ig (**Figure S4**).

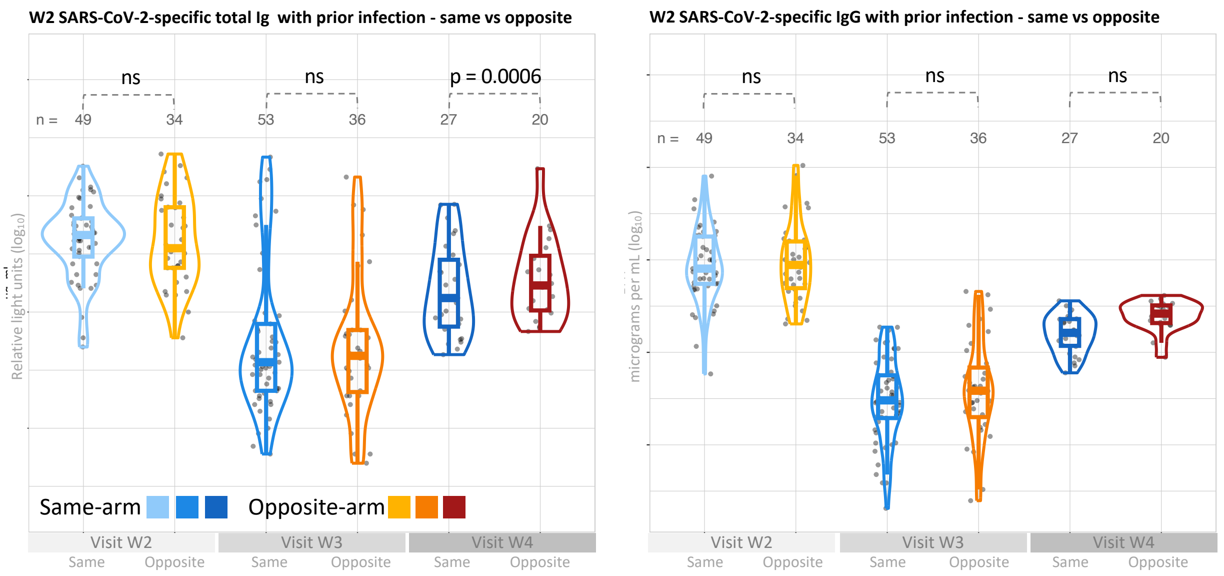


**Figure S4. SARS-CoV-2-specific total Ig and IgG at W2, W3 and W4, by arm group in participants with natural infection prior to W3.** Analysis of the effect of arm selection at V2 in from among 139 participants with one or more evaluable timepoints and a history of natural COVID-19 infection between V1 and W3. **Left panel:** Total SARS-CoV2 specific Ig titer ($\log_{10}$ RLU) by Lumit assay. **Right panel:** SARS-CoV-2-specific IG by ELISA ($\log_{10}$ ug/ml) by ELISA at W3 and W4 in persons receiving V2 in the same arm or opposite arm relative to V1. Vaccination and visit timepoints are represented by "V_", "W_" respectively (see Figure 1). Significance determined by two-tailed T-test. **Interpretation**: Among those with a history of natural infection, contralateral vaccination at V2 is associated with a trend towards higher antibody titers at late timepoints W3 and W4, compared with ipsilateral boosting.

**Arm selection at vaccine dose V3 (Enrolled Analysis Group):** To examine the effect of arm selection at vaccine dose 3 on immune responses, we sorted all study participants into four groups based on patterns of arm selection across 3 doses: RRR and LLL ("Same-Same"), RLR and LRL ("Opposite-Opposite"), RRL and LLR ("Same-Opposite"), and RLL and LRR ("Opposite-Same"). SARS-CoV-2-specific total Ig and IgG at W4 were compared across groups. Here, OS and OO groups have greater responses than SS and SO groups. This trend was significant for total Ig, but did not reach significance for IgG. These results suggest that the long-term advantage in immune responses resulting from arm alternation

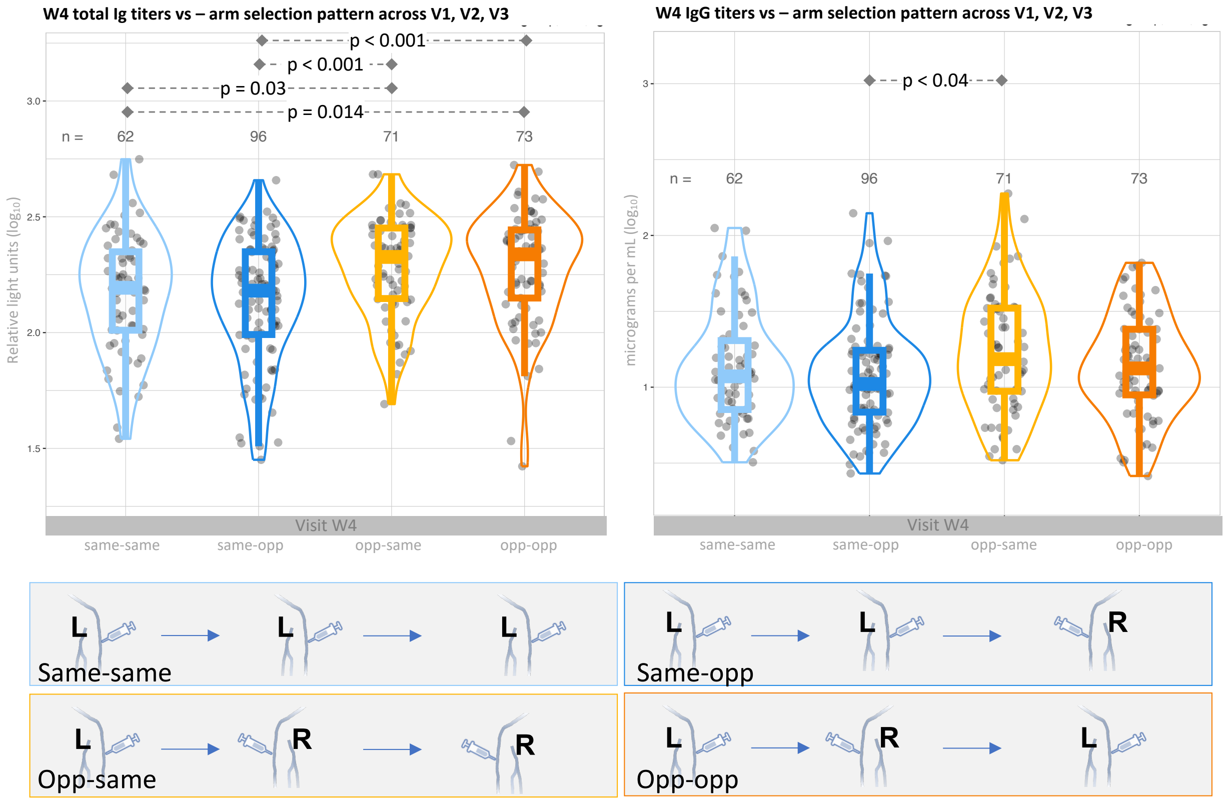


**Figure S5. SARS-CoV-2-specific antibody titers by arm selection across V1, V2 and V3 (Enrolled Analysis Group).** Immune responses at W4 in individuals vaccinated at V1, V2, and V3 compared by arm usage patterns across three vaccinations (same-same, same-opp, opp-same and opp-opp). **Left panel:** Total SARS-CoV-2-specifc Ig (Lumit assay). **Right panel:** IgG (ELISA). Y –axis: antibody titers, $Log_{10}$ scale. Only significant comparisons are shown. Vaccination and visit timepoints are represented by "V_", "W_" respectively (see Figure 1). Significance determined by two-tailed T-test. **Interpretation**: The opp-same and opp-opp groups show significantly higher SARS-CoV2-specific Ig levels than the remaining groups and a similar but non-significant trend is apparent for IgG levels, suggesting that arm selection at V1 and V2 are most influential in conferring contralateral vaccination improvement, while site selection at the third vaccination has little impact.

in this setting pertain to the first two vaccine doses, while arm selection at the third dose has little effect (**Figure S5**).

**Time dependence of contralateral advantage at W2 (Unselected Analysis Group):** To define the time-dependence of immunologic improvements resulting from contralateral arm vaccination, we analyzed SARS-CoV-2-specific total Ig and IgG at W2 as a function of time elapsed since V2. While this study was not designed to assess this variable, the large number of participants presenting at a range of times after V2 offered the possibility of insight into this question in a preliminary cross-sectional analysis. The starting hypothesis is that shortly after V2 vaccination, same-arm boost recipients would show an advantage due to stimulation of secondary germinal centers within the ipsilateral lymph node group, while boosting on the contralateral side would initiate de-novo responses on the contralateral side during this timeframe. However, at subsequent timepoints an overall advantage of contralateral boosting would emerge, with a cross-over time determined in part by the time required for emergence of antibodies derived from the contralateral LN group. Using locally-weighted polynomial regression (LOESS) we found that antibody response patterns in these two groups were consistent with this expectation, with an apparent cross-over time within the first 3 weeks after V2 (**Figure S6**).

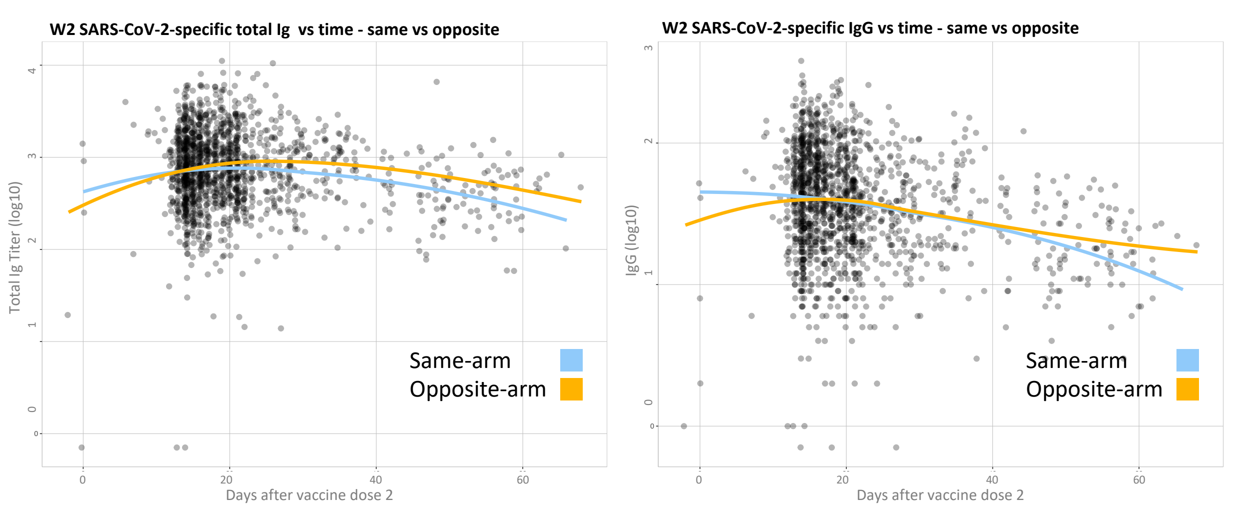


**Figure S6. Time dependence of contralateral advantage (Unselected Analysis Group).** Local regression plot of SARS-CoV-2-specific serum antibodies at visit W2 versus time since vaccine dose 2. **Left panel:** Total SARS-CoV2 specific Ig titer ($log_{10}$ RLU) by Lumit assay. **Right panel:** SARS-CoV-2-specific IG by ELISA ($log_{10}$ ug/ml). **Interpretation**: Local regression (LOESS) lines are consistent with a transition from higher levels with ipsilateral boosting to higher levels with contralateral boosting, with a crossover time at approximately 2-3 weeks after vaccine dose 2.

**Effect of arm selection at V2 on antibody quality (Matched Pair Group):** The improved neutralization titers observed at W4 in those receiving boosting in the opposite arm at V2 could be explained by higher antibody titers or improved antibody affinity. We therefore analyzed the ratio of neutralization to binding antibodies (either total Ig, or IgG) at W3 and W4. Comparisons were made with respect to neutralization against D146G (early virus) and B.1.1.529 (Omicron variant). In these analyses we initially see a lower ratio at W3 in the opposite arm group, and infer lower antibody affinity at this timepoint. However, the trend reverses by W4, suggesting that in addition to higher total antibody titers (e.g. Figure S3), affinity maturation (as reflected in the calculated ratio) also begins to contribute to improved neutralization titers between W3 and W4 in those receiving opposite-arm boosting (**Figure S7**).

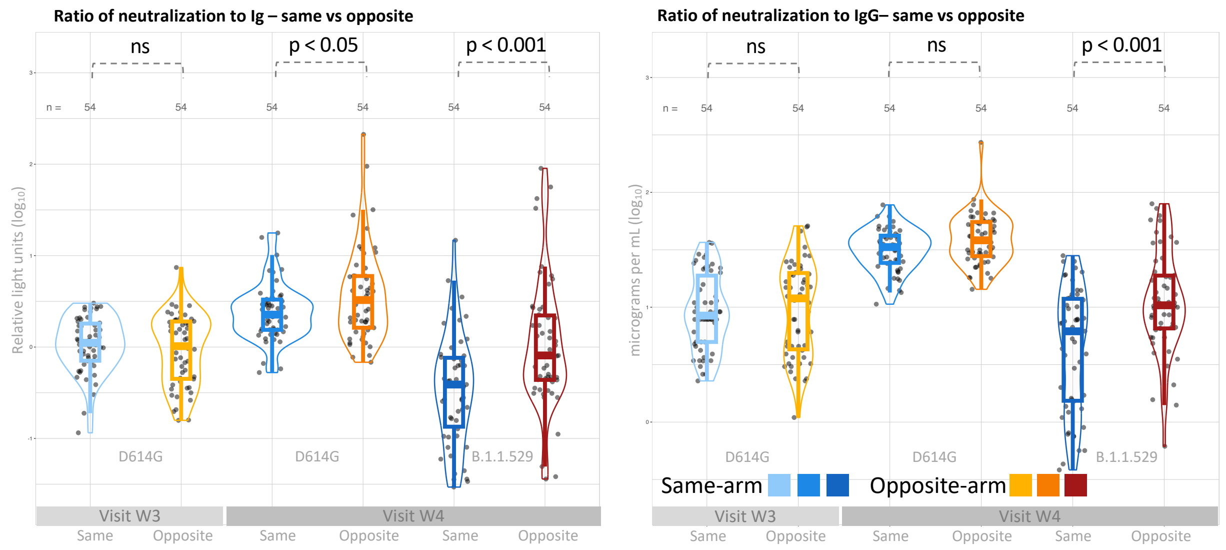


**Figure S7. Antibody quality by arm group (Matched Pair Group) –** Ratio of neutralization to binding antibody levels in serum as a function of arm selection group (opposite or same) at V2. **Left panel**: neutralization vs total Ig (Lumit). **Right panel**: neutralization vs IgG (ELISA). Y axis = neutralization to immunoglobulin ratio ($log_{10}$ scale) for pseudoviruses representing early COVID-19 strain D146G at W3 (left, each panel), D146G at visit W4 (center, each panel), and late COVID-19 strain B.1.1529 at visit W4 (right, each panel). Vaccination and visit timepoints are represented by “V_”, “W_” respectively (see Figure 1). Significance determined by two-tailed T-test. **Interpretation**: The ratio of neutralization to serum Ig and IgG rises from W3 to W4, demonstrating expected antibody affinity maturation against homologous early strain D146G. This ratio rises to a higher level in the opposite arm group (in this series, significant only for Ig), and is also greater against future epidemic virus B.1.1.529, indicating that contralateral vaccination at V2 results in greater affinity and neutralization breadth.
